# Correction: In-chain functionalized syndiotactic 1,2-polybutadiene by a Ziegler–Natta iron(iii) catalytic system

**DOI:** 10.1039/d0ra90133d

**Published:** 2021-02-02

**Authors:** Shanshan Liang, Huaqiang Zhang, Rixin Cong, Heng Liu, Feng Wang, Yanming Hu, Xuequan Zhang

**Affiliations:** College of Material Science and Engineering, Shenyang University of Chemical Technology Shenyang 110142 China; CAS Key Laboratory of High-Performance Synthetic Rubber and Its Composite Materials, Changchun Institute of Applied Chemistry, Chinese Academy of Sciences 5625 Renmin Street Changchun 130022 P. R. China fengwang@ciac.ac.cn xqzhang@ciac.ac.cn; Lanzhou Petrochemical Research Center, Petrochemical Research Institute PetroChina Lanzhou 730060 China; School of Chemical Engineering, Changchun University of Technology Changchun Jilin China

## Abstract

Correction for ‘In-chain functionalized syndiotactic 1,2-polybutadiene by a Ziegler–Natta iron(iii) catalytic system’ by Shanshan Liang *et al.*, *RSC Adv.*, 2019, **9**, 33465–33471, DOI: 10.1039/C9RA06499K.

The authors regret that an incorrect grant number was shown in the acknowledgements section of the published article. The corrected section should read:

This work was supported by the National Natural Science Foundation of China (No. 21801236), PetroChina Company Limited (2018B-2707), Joint Funds of the National Natural Science Foundation of China (U1862206) and Jilin Provincial Science and Technology Development program (20190103122JH).

The Royal Society of Chemistry apologises for these errors and any consequent inconvenience to authors and readers.

## Supplementary Material

